# Nerve Stimulation Monitoring Devices in Radical Prostatectomy: A Narrative Review

**DOI:** 10.7759/cureus.105860

**Published:** 2026-03-25

**Authors:** Vasileios Panagopoulos, Evangelia Dimitrakopoulou, Stamatina Vlachou

**Affiliations:** 1 5th Urology Department, Iaso General Hospital, Athens, GRC; 2 General Surgery Department, General Oncological Hospital of Kifissia "Agioi Anargyroi", Athens, GRC

**Keywords:** cavernous nerve, erectile function, intraoperative neuromonitoring, nerve stimulation, neurovascular bundle, radical prostatectomy, urinary continence

## Abstract

Preservation of neurovascular bundles during radical prostatectomy is crucial for postoperative erectile function and urinary continence. Intraoperative nerve stimulation and neurophysiological monitoring devices have been developed to assist surgeons in identifying and preserving cavernous nerves, aiming to improve functional outcomes. A comprehensive review of the literature was conducted using the PubMed database, focusing on studies published within the last 10-15 years addressing intraoperative nerve stimulation and monitoring during radical prostatectomy. Multiple monitoring techniques have been described, including intracorporeal pressure measurement, penile tumescence monitoring, laser Doppler flowmetry, urethral pressure profilometry, and electromyography. While these methods are technically feasible and provide real-time functional feedback, evidence demonstrating a consistent and statistically significant improvement in postoperative erectile function or urinary continence remains limited. Current data do not support the routine use of intraoperative nerve stimulation monitoring devices during radical prostatectomy. Surgical experience, meticulous anatomical dissection, and minimal tissue manipulation remain the most important determinants of functional outcomes. Further large-scale, standardized prospective studies are required.

## Introduction and background

Prostate cancer is a major cause of cancer-related morbidity and mortality among men worldwide. The global annual incidence is estimated at approximately 1.5 million cases, with more than 350,000 deaths per year [1,2]. Despite advances in radiotherapy and systemic treatments, radical prostatectomy remains a cornerstone in the management of localized prostate cancer and is also incorporated into multimodal strategies for selected patients with locally advanced disease, according to contemporary European Association of Urology (EAU) guidelines [3-7].

Regardless of the surgical approach (open, laparoscopic, or robotic), preservation of the neurovascular bundles (NVBs) is critical for postoperative functional outcomes. Nerve-sparing techniques vary (intrafascial, interfascial, and extrafascial dissection planes), with increasing preservation associated with improved erectile function but potentially a higher risk of positive surgical margins in locally advanced disease [8-10].

Intraoperative identification and preservation of NVBs remain technically challenging due to anatomical variability and their complex, distributed architecture. Rather than forming a single discrete bundle, cavernous nerves are organized in a network extending posterolaterally and, in some cases, anteriorly around the prostate. This variability contributes to the difficulty of precise nerve identification and preservation during surgery.

To address these challenges, intraoperative nerve stimulation and neurophysiological monitoring technologies have been introduced as adjuncts to visual anatomical identification. These techniques aim to provide real-time functional assessment of nerve integrity and potentially improve postoperative erectile function and urinary continence. However, their clinical utility and impact on long-term outcomes remain uncertain.

## Review

Materials and methods

This manuscript is a narrative review of the literature on intraoperative nerve stimulation and neuromonitoring during radical prostatectomy. A structured literature search was performed in PubMed using combinations of the terms “radical prostatectomy”, “nerve stimulation”, “cavernous nerve”, “neurovascular bundle”, and “intraoperative monitoring”. The search focused primarily on studies published within the last 15 years, while selected earlier landmark studies were included for historical context.

Studies were selected based on their relevance to intraoperative neuromonitoring techniques and reported functional outcomes (erectile function and urinary continence). No formal systematic inclusion/exclusion criteria or meta-analysis were applied, consistent with the narrative nature of the review. The evidence was synthesized qualitatively with emphasis on methodological limitations and clinical applicability.

Neuroanatomy

The NVBs are a complex network of autonomic fibers essential for erectile function and contributing to urinary continence. Their identification remains a key challenge during radical prostatectomy, even with enhanced visualization in minimally invasive approaches.

Rather than forming a single discrete structure, cavernous nerves are distributed in a variable, predominantly posterolateral pattern around the prostate, with extension toward the apex and, in some cases, anteriorly. This dispersed architecture contributes to the difficulty of precise intraoperative identification and preservation.

The NVBs lie within a fascial plane defined by Denonvilliers’ fascia, the prostatic fascia, and the lateral pelvic fascia. Critical surgical steps associated with potential nerve injury include bladder neck dissection, seminal vesicle mobilization, pedicle control, and apical dissection. In addition to mechanical trauma, thermal injury from energy devices represents an important mechanism of nerve damage [11,12].

Devices and monitoring techniques

Despite the recognized importance of preserving the NVB, the existing protocols and techniques are relatively limited and not well standardized. Irrespectively of the device used, the main principle is the identification and mapping of the NVBs through neural stimulation. Based on this rationale, intraoperative neurostimulation and monitoring have been proposed.

According to the published data, stimulation performed with a bipolar probe may result in physiological responses such as increased penile blood flow, intracavernosal pressure and pressure changes, bladder neck contraction, urethral pressure increase, and activation of pelvic floor and external anal sphincter musculature. Intraoperatively, the probe is typically applied at different sites along the prostate pedicles, Denonvilliers’ fascia, and the anatomical course of the pudendal nerve. Commonly reported settings include 10-30 mA with a pulse duration of 0.22-0.5 ms for 20-40 s [12-14].

The most commonly described device is the CaverMap Surgical Aid (Uromed Corporation, Boston, MA, USA), which records intraoperative changes in penile tumescence [15-19]. In addition, other techniques such as urethral pressure profilometry, laser Doppler flowmetry of penile blood flow, and corpus cavernosum electromyography have been proposed [20-22]. More recently, robotic-compatible stimulation probes and real-time signal processing systems have been introduced, allowing integration with minimally invasive platforms.

Despite the variety of available modalities (Table 1), their clinical application remains limited. Most of the published studies are small, non-comparative, and in many cases performed in open surgical settings, which limits their applicability to contemporary robotic surgery. While these techniques provide real-time feedback, their impact on postoperative erectile function and urinary continence remains uncertain, as also reflected in the functional outcomes summarized in Table 2.

**Table 1:** Comparative overview of intraoperative nerve stimulation and monitoring techniques during radical prostatectomy. EMG: electromyography

Study	Surgical approach	Monitoring modality	Patients (n)	Main outcome	Key limitations
Rehman et al. [14]	Open	Intracorporeal pressure	14	Correlation with erectile function	Small sample
Walsh et al. [18]	Open	CaverMap tumescence	129	Sensitivity 87%, specificity 54%	Low specificity
Axelson et al. [20]	Open	Laser Doppler flowmetry	20	Increased penile blood flow	Limited functional benefit
Nelson et al. [21]	Open	Urethral pressure monitoring	8	Improved sphincter activation	Feasibility only
Song et al. [22]	Robotic	Cavernosal EMG	30	Feasible nerve mapping	Phase I/II

**Table 2:** Functional outcomes reported in studies using intraoperative nerve stimulation during radical prostatectomy. ICP: intracavernosal pressure; EMG: electromyography

Study	Monitoring technique	Follow-up	Erectile function outcome	Continence outcome
Rehman et al. [14]	ICP monitoring	12 mo	Improved erections if ICP > 50 cmH_2_O	Not assessed
Klotz [19]	CaverMap	12 mo	No significant long-term benefit	Not assessed
Axelson et al. [20]	Laser Doppler	6-12 mo	Minimal improvement	Not assessed
Nelson et al. [21]	Urethral pressure	Early postop	Not assessed	Improved sphincter activation
Song et al. [22]	Cavernosal EMG	12 mo	Feasible, trend to improvement	Comparable to controls

Discussion

As far as our research indicates, the first data concerning electrical stimulation of cavernosal nerves during radical prostatectomy were published by Rehman et al. [14] in 1999. In that study, 14 patients who underwent nerve-sparing open retropubic radical prostatectomy were included, and a direct association between penile intracorporeal pressure and postoperative erectile function was reported. According to the outcomes, preservation of nerve integrity and achievement of intracorporeal pressure greater than 50 cmH_2_O were associated with adequate postoperative erections [14]. During the last decades, accumulating knowledge on neurophysiology and on periprostatic vascular and neural tissues has contributed to improved postoperative continence and sexual function through enhanced neural-vascular awareness and preservation. However, many aspects of pelvic neurophysiology remain incompletely understood [12].

As already mentioned, the CaverMap Surgical Aid is probably the most commonly used device for neural stimulation and penile tumescence monitoring. Despite its relatively simple use, precise anatomical identification of the cavernous nerves remains challenging, as various perioperative factors may alter the surgical field [15]. According to a multi-institutional study conducted by Walsh et al. [18], the sensitivity of the device exceeded 87%, whereas its specificity in localizing the NVBs was relatively low (54%), which largely explains its limited adoption in routine practice [18]. Another potential cause of reduced precision is the relatively bulky stimulator probe [17]. In terms of potency and erectile outcomes, reported results remain inconclusive. As shown in Table 2, some studies suggest improvement in early postoperative erectile responses, while the overall benefit in long-term sexual function remains marginal [16,17,19]. Most authors agree that meticulous anatomical dissection, detailed knowledge of periprostatic anatomy, and minimal manipulation of surrounding tissues are the primary determinants of functional outcomes after radical prostatectomy, irrespective of the adjunctive technologies employed [23]. This is in line with broader evidence suggesting that postoperative potency is influenced mainly by nerve-sparing quality, surgical technique, and surgeon experience rather than adjunctive intraoperative technologies [24].

Similar findings were reported in a randomized, phase II/III, multicenter, single-blinded study conducted by Klotz et al. [16], which included a relatively small cohort of 61 patients [16,19]. In an attempt to better understand and monitor pelvic neural function, penile laser Doppler blood flowmetry has also been proposed. Proper electrical stimulation of the NVBs was shown to increase penile blood flow and girth; however, long-term postoperative erectile outcomes remained disappointing [20].

Beyond erectile function, the NVBs also play a crucial role in urinary continence. Nelson et al. [21] conducted a feasibility study involving eight patients undergoing nerve-sparing open radical prostatectomy with concomitant nerve stimulation and urodynamic assessment. Electrical stimulation resulted in urethral sphincter activation and increased urethral pressure, suggesting that adequate preservation of neurovascular structures may contribute to improved continence rates [21]. Nevertheless, as reflected in Table 2, these findings remain limited and do not establish a consistent improvement in postoperative continence.

These techniques and devices have also been applied in minimally invasive settings, including laparoscopic and robot-assisted radical prostatectomy [13,22]. Notably, Song et al. [22] described the use of corpus cavernosum electromyography during neurovascular stimulation in a robotic-assisted series. Despite encouraging feasibility results, all such studies are limited by small sample size [22]. In addition, although these technologies have also been explored in minimally invasive and robotic settings, the majority of the available literature still derives from open surgery, which limits the applicability of these findings to contemporary practice [25].

Importantly, the presence of an intraoperative physiological response does not necessarily translate into improved long-term functional outcomes. Several factors may explain this discrepancy. Surgeon experience and surgical volume remain key determinants of outcomes, while the quality of nerve-sparing technique appears to play a more significant role than adjunctive technologies. In addition, thermal injury from energy devices and excessive tissue manipulation may negatively affect nerve integrity despite apparent anatomical preservation.

Another important limitation is the variability in outcome assessment. Validated tools for erectile function and urinary continence are not consistently used, making comparisons between studies difficult. Furthermore, most studies do not provide robust comparative statistical analysis, and measures such as odds ratios, hazard ratios, or adequately powered comparisons are rarely reported. Although preservation of the NVBs remains the most critical surgical factor for postoperative sexual and urinary function, none of the published studies have demonstrated a clear or statistically significant functional advantage attributable solely to the use of neurostimulation devices [19].

The available literature is further limited by small sample sizes, lack of appropriate control groups, heterogeneity in stimulation protocols, outcome definitions, and variability in follow-up duration. These methodological constraints substantially restrict the generalizability of the existing evidence and underscore the need for larger, well-designed prospective trials incorporating standardized neuromonitoring protocols and clinically meaningful functional endpoints.

Overall, although intraoperative nerve stimulation and monitoring techniques are technically feasible and provide real-time feedback, the findings summarized in Table 2 do not demonstrate a consistent or clinically meaningful improvement in postoperative functional outcomes. Their role, therefore, remains limited, and functional results continue to depend primarily on surgical expertise, anatomical knowledge, and careful tissue handling.

## Conclusions

Intraoperative nerve stimulation and neurophysiological monitoring during radical prostatectomy remain technically feasible and conceptually attractive tools for enhancing nerve-sparing surgery. However, current evidence does not demonstrate a clear, reproducible functional benefit in terms of erectile function or urinary continence. Larger, well-designed prospective trials incorporating standardized stimulation protocols and contemporary robotic platforms are required before these technologies can be routinely recommended.
